# A Bat Algorithm with Mutation for UCAV Path Planning

**DOI:** 10.1100/2012/418946

**Published:** 2012-12-27

**Authors:** Gaige Wang, Lihong Guo, Hong Duan, Luo Liu, Heqi Wang

**Affiliations:** ^1^Changchun Institute of Optics, Fine Mechanics and Physics, Chinese Academy of Sciences, Changchun 130033, China; ^2^Graduate School of Chinese Academy of Sciences, Beijing 100039, China; ^3^School of Computer Science and Information Technology, Northeast Normal University, Changchun 130117, China

## Abstract

Path planning for uninhabited combat air vehicle (UCAV) is a complicated high dimension optimization problem, which mainly centralizes on optimizing the flight route considering the different kinds of constrains under complicated battle field environments. Original bat algorithm (BA) is used to solve the UCAV path planning problem. Furthermore, a new bat algorithm with mutation (BAM) is proposed to solve the UCAV path planning problem, and a modification is applied to mutate between bats during the process of the new solutions updating. Then, the UCAV can find the safe path by connecting the chosen nodes of the coordinates while avoiding the threat areas and costing minimum fuel. This new approach can accelerate the global convergence speed while preserving the strong robustness of the basic BA. The realization procedure for original BA and this improved metaheuristic approach BAM is also presented. To prove the performance of this proposed metaheuristic method, BAM is compared with BA and other population-based optimization methods, such as ACO, BBO, DE, ES, GA, PBIL, PSO, and SGA. The experiment shows that the proposed approach is more effective and feasible in UCAV path planning than the other models.

## 1. Introduction

Uninhabited combat aerial vehicle (UCAV) is one of inevitable trends of the modern aerial weapon equipment which develop in the direction of unmanned attendance and intelligence. Research on UCAV directly affects battle effectiveness of the air force and is fatal and fundamental research related to safeness of a nation. Path planning and trajectory generation is one of the key technologies in coordinated UCAV combatting. The flight path planning in a large mission area is a typical large scale optimization problem; a series of algorithms have been proposed to solve this complicated multiconstrained optimization problem, such as differential evolution [1], biogeography-based optimization [2, 3], genetic algorithm [4], ant colony algorithm [5] and its variant [6, 7], cuckoo search [8, 9], chaotic artificial bee colony [10], firefly algorithm [11, 12], and intelligent water drops optimization [13]. However, those methods can hardly solve the contradiction between the global optimization and excessive information. 

In 1995, Storn and Price firstly proposed a novel evolutionary algorithm (EA): differential evolution (DE) [14], which is a new heuristic approach for minimizing possibly nonlinear and nondifferentiable continuous space functions. It converges faster and with more certainty than many other acclaimed global population-based optimization methods. This new method requires few control variables, which makes DE more robust and easy to use and lend itself very well to parallel computation.

First presented in [15], the bat-inspired algorithm or bat algorithm (BA) is a metaheuristic search algorithm, inspired by the echolocation behavior of bats with varying pulse rates of emission and loudness. The primary purpose of a bat's echolocation is to act as a signal system to sense distance. 

However, in the field of path planning for UCAV, no application of BA algorithm exists yet. In this paper, we use an original BA and an improved modified BA algorithm to solve UCAV path planning problem. Here, we add mutation operation in DE between bats to propose a new metaheuristic algorithm according to the principle of BA, and then an improved BA algorithm is used to search the optimal or suboptimal route with complicated multiconstraints. To investigate the feasibility and effectiveness of our proposed approach, it is compared with BA and other population-based optimization methods, such as ACO, BBO, DE, ES, GA, PBIL, PSO, and SGA under complicated combating environments. The simulation experiments indicate that our hybrid metaheuristic method can generate a feasible optimal route for UCAV more effectively than other population-based optimization methods. 

The remainder of this paper is structured as follows.  Section 2 describes the mathematical model in UCAV path planning problem. Subsequently, the principle of the basic BA is explained in Section 3, and then an improved BA with mutation for UCAV path planning is presented in Section 4 and the detailed implementation procedure is also described in this section. The simulation experiment is conducted in Section 5. Finally, Section 6 concludes the paper and discusses the future path of our work.

## 2. Mathematical Model in UCAV Path Planning 

Path planning for UCAV is a new low altitude penetration technology to achieve the purpose of terrain following and terrain avoidance and flight with evading threat, which is a key component of mission planning system [16]. The goal for path planning is to calculate the optimal or suboptimal flight route for UCAV within the appropriate time, which enables the UCAV to break through the enemy threat environments, and self-survive with the perfect completion of mission. In our work, we use the mathematical model in UCAV path planning in [1], which is described as follows.

### 2.1. Problem Description

**Figure 1:**
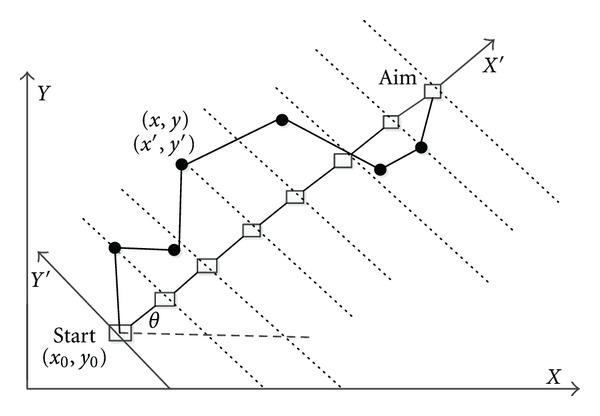
Coordinates transformation relation.

Path planning for UCAV is the design of optimal flight route to meet certain performance requirements according to the special mission objective and is modeled by the constraints of the terrain, data, threat information, fuel, and time. In this paper, firstly the route planning problem is transformed into a *D*-dimensional function optimization problem (Figure 1).

In Figure 1, we transform the original coordinate system into new coordinate whose horizontal axis is the connection line from starting point to target point according to transform expressions shown as (1), where the point (*x*, *y*) is coordinate in the original ground coordinate system *O*_*XY*_; the point (*x*′, *y*′) is coordinate in the new rotating coordinate system *O*_*X*′*Y*′_; *θ* is the rotation angle of the coordinate system. One has

(1) $$\begin{array}{c} \theta =\arcsin\frac{y_{2}-y_{1}}{\left|\vec{AB}\right|},\\ \left(\begin{array}{c} x\\ y \end{array}\right)=\left(\begin{array}{c} \begin{array}{cc} \cos\theta & \sin\theta \end{array}\\ \begin{array}{cc} -\sin\theta & \cos\theta \end{array} \end{array}\right)•\left(\begin{array}{c} x^{\prime }\\ y^{\prime } \end{array}\right)+\left(\begin{array}{c} x_{1}\\ y_{1} \end{array}\right). \end{array}$$

Then, we divide the horizontal axis *X*′ into *D* equal partitions and then optimize vertical coordinate *Y*′ on the vertical line for each node to get a group of points composed by vertical coordinate of *D* points. Obviously, it is easy to get the horizontal abscissas of these points. We can get a path from start point to end point through connecting these points together, so that the route planning problem is transformed into a *D*-dimensional function optimization problem.

### 2.2. Performance Indicator

A performance indicator of path planning for UCAV mainly contains the completion of the mandate of the safety performance indicator and fuel performance indicator, that is, indicators with the least threat and the least fuel.

Minimum of performance indicator for threat

(2) $$\begin{array}{c} \min J_{f}=\int_{0}^{L}w_{t}dl,\;L\;\;\text{is}\;\text{the}\;\text{length}\;\text{of}\;\text{the}\;\text{path}. \end{array}$$

Minimum of performance indicator for fuel

(3) $$\begin{array}{c} \min J_{f}=\int_{0}^{L}w_{f}dl,\;L\;\;\text{is}\;\text{the}\;\text{length}\;\text{of}\;\text{the}\;\text{path}. \end{array}$$

Then the total performance indicators for UCAV route

(4) $$\begin{array}{c} \min J=kJ_{t}+\left(1-k\right)J_{f}, \end{array}$$

where *w*_*t*_ is the threat cost for each point on the route; *w*_*f*_ is fuel cost for each point on the path which depends on path length (in this paper, *w*_*f*_ ≡ 1); *k* ∈ [0,1] is balanced coefficient between safety performance and fuel performance, whose value is determined by the special task UCAV performing; that is, if flight safety is of highly vital importance to the task, then we choose a larger *k*, while if the speed is critical to the aircraft task, then we select a smaller *k*.

### 2.3. Threat Cost

When the UCAV is flying along the path *L*_*ij*_, the total threat cost generated by *N*_*t*_ threats is calculated as follows:

(5) $$\begin{array}{c} w_{t,L_{ij}}=\int_{0}^{L_{ij}}\sum_{k=1}^{N_{t}}\frac{t_{k}}{{\left[{\left(x-x_{k}\right)}^{2}+{\left(y-y_{k}\right)}^{2}\right]}^{2}}dl. \end{array}$$

**Figure 2:**
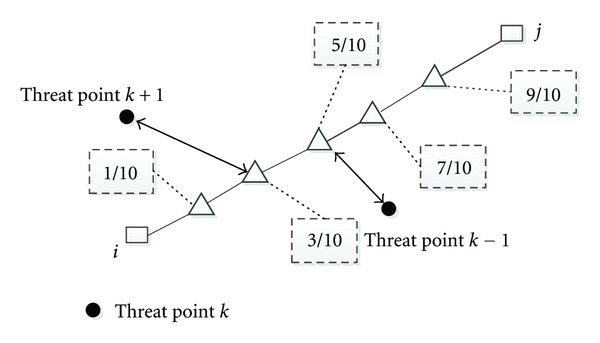
Modeling of the UCAV threat cost [6].

To simplify the calculations (as shown in Figure 2), each path segment is discretized into five subsegments and the threat cost is calculated on the end of each subsegment. If the distance from the threat point to the end of each subsegment is within threat radius, we can calculate the responding threat cost according to

(6) $$\begin{array}{c} w_{t,L_{ij}}=\frac{L_{{}_{ij}}^{5}}{5}\sum_{k=1}^{N_{t}}t_{k}\left(\frac{1}{d_{0.1,k}^{4}}+\frac{1}{d_{0.3,k}^{4}}+\frac{1}{d_{0.5,k}^{4}}+\frac{1}{d_{0.7,k}^{4}}+\frac{1}{d_{0.9,k}^{4}}\right), \end{array}$$

where *L*_*ij*_ is the length of the subsegment connecting node *i* and node*j*; *d*_0.1,*k*_ is the distance from the 1/10 point on the subsegment *L*_*ij*_ to the *k*th threat; *t*_*k*_ is threat level of the *k*th threat.

As fuel cost related to flight length, we can consider  *w*_*f*_ = *L*, for simplicity, and fuel cost of each edge can be expressed by *w*_*f*,*L*_*ij*__ = *L*_*ij*_.

## 3. Bat Algorithm (BA) 

The bat algorithm is a new swarm intelligence optimization method, in which the search algorithm is inspired by social behavior of bats and the phenomenon of echolocation to sense distance. 

### 3.1. Mainframe of BA

In [17], for simplicity, bat algorithm is based on idealizing some of the echolocation characteristics of bats, which are following approximate or idealized rules.

1.  All bats apply echolocation to sense distance, and they always “know” the surroundings in some magical way.
2.  Bats fly randomly with velocity *v*_*i*_ and a fixed frequency *f*_min⁡_ at position *x*_*i*_, varying wavelength *λ*, and loudness *A*_0_ to hunt for prey. They can spontaneously accommodate the wavelength (or frequency) of their emitted pulses and adjust the rate of pulse emission *r* ∈ [0, 1], depending on the proximity of their target.
3.  Although the loudness can change in different ways, it is supposed that the loudness varies from a minimum constant (positive) *A*_min⁡_ to a large *A*_0_.

Based on these approximations and idealization, the basic steps of the bat algorithm (BA) can be described as shown in Algorithm 1. In BA, each bat is defined by its position *x*_*i*_^*t*^, velocity *v*_*i*_^*t*^, frequency *f*_*i*_, loudness *A*_*i*_^*t*^, and the emission pulse rate *r*_*i*_^*t*^ in a *d*-dimensional search space. The new solutions *x*_*i*_^*t*^ and velocities *v*_*i*_^*t*^ at time step *t* are given by

(7) $$\begin{array}{c} f_{\text{i}}=f_{\min}+\left(f_{\max}-f_{\min}\right)\beta ,\\ v_{i}^{t}=v_{i}^{t-1}+\left(x_{i}^{t}-x_{*}\right)f_{i},\\ x_{i}^{t}=x_{i}^{t-1}+v_{i}^{t}, \end{array}$$

where *β* ∈ [0, 1] is a random vector drawn from a uniform distribution. Here **x**_∗_ is the current global best location (solution) which is located after comparing all the solutions among all the *n* bats. Generally speaking, depending on the domain size of the problem of interest, the frequency *f* is assigned to *f*_min⁡_ = 0 and *f*_max⁡_ = 100 in practical implementation. Initially, each bat is randomly given a frequency which is drawn uniformly from [*f*_min⁡_, *f*_max⁡_]. 

For the local search part, once a solution is selected among the current best solutions, a new solution for each bat is generated locally using random walk

(8) $$\begin{array}{c} x_{\text{new}}\;=\;x_{\text{old}}\;+\;\varepsilon A^{t}, \end{array}$$

where *ε* ∈ [−1, 1] is a scaling factor which is a random number, while *A*_*t*_ = 〈*A*_*i*_^*t*^〉 is the average loudness of all the bats at time step *t*. 

The updates of the velocities and positions of bats have some similarity to the procedure in the standard particle swarm optimization [18] as *f*_*i*_ in essence controls the pace and range of the movement of the swarming particles. To some degree, BA can be considered as a balanced combination of the standard particle swarm optimization and the intensive local search controlled by the loudness and pulse rate. 

Furthermore, the loudness *A*_*i*_ and the rate *r*_*i*_ of pulse emission update accordingly as the iterations proceed as shown in

(9) $$\begin{array}{c} A_{i}^{t+1}=\alpha A_{i}^{t},\;\;r_{i}^{t+1}=r_{i}^{0}\left[1-\exp\left(-\gamma t\right)\right], \end{array}$$

where *α* and *γ* are constants. In essence, *α* is similar to the cooling factor of a cooling schedule in the simulated annealing [19]. For simplicity, we set *α* = *γ* = 0.9 in this work.

### 3.2. Algorithm BA for UCAV Path Planning

In BA, the standard ordinates are inconvenient to solve UCAV path planning directly. In order to apply BA to UCAV path planning, one of the key issues is to transform the original ordinate into rotation ordinate by (1).

Fitness of bat *i* at position *x*_*i*_ is determined by the threat cost by (4), and the smaller the threat cost, the smaller the fitness of bat *i* at position *x*_*i*_. Each bat is encoded by *D*-dimensional deciding variables. And then, we use BA to optimize the path planning to get the best solution that is optimal flight route for UCAV. At last, the best solution is inversely converted to the original ordinates and output. The algorithm BA for UCAV path planning is shown as Algorithm 2.

## 4. Bat Algorithm with Mutation (BAM) 

The differential evolution (DE) algorithm, proposed by Storn and Price [14], is a simple evolutionary algorithm (EA) which generates new candidate solutions by combining the parent individual and a few other individuals of the same population. A candidate substitutes the parent only if it has better fitness. This is a rather greedy selection scheme which often overtakes traditional EAs. Advantages of DE are easy implementation, simple structure, speed, and robustness.

In general, the standard DE algorithm is adept at exploring the search space and locating the region of global optimal value, but it is not relatively good at exploiting solution. On the other hand, standard BA algorithm is usually quick at the exploitation of the solution though its exploration ability is relatively poor. Therefore, in this paper, a hybrid metaheuristic algorithm by inducing mutation in differential evolution into bat algorithm, so-called bat algorithm with mutation (BAM), is used to solve the path planning for UCAV. The difference between BAM and DE is that the mutation operator is used to improve the original BA generating new solution for each bat with a probability 1 − *r* originally using random walk. In this way, this method can explore the new search space by the mutation of the DE algorithm and exploit the population information with BA and therefore can overcome the lack of the exploitation of the DE algorithm. In the following, we will show the algorithm BAM which is a variety of DE and BA. Firstly, we describe a mainframe of BAM, and then an algorithm BAM for UCAV path planning is shown. 

### 4.1. Mainframe of BAM

The critical operator of BAM is the hybrid differential evolution mutation operator, which composes the mutation operation in differential evolution with the BA. The core idea of the proposed hybrid mutation operator is based on two considerations. First, poor solutions can take in many new used features from good solutions. Second, the mutation operator of DE can improve the exploration of the new search space. In this way, we composed mutation operation into BAM which modifies the solutions with poor fitness in order to add diversity of the population to improve the search efficiency. 

For bat algorithm, as the search relies entirely on random walks, a fast convergence cannot be guaranteed. Described here for the first time, a main modification of adding mutation operator is made to the BA, including two minor modifications, which are made with the aim of speeding up convergence, thus making the method more practical for a wider range of applications but without losing the attractive features of the original method. 

The first modification is that we use fixed frequency *f* and loudness *A* instead of various frequency *f*_*i*_ and *A*_*i*_^*t*^. Similar to BA, in BAM, each bat is defined by its position  *x*_*i*_^*t*^, velocity *v*_*i*_^*t*^, the emission pulse rate *r*_*i*_^*t*^, the fixed frequency *f*, and loudness *A* in a *d*-dimensional search space. The new solutions *x*_*i*_^*t*^ and velocities *v*_*i*_^*t*^ at time step *t* are given by

(10) $$\begin{array}{c} v_{i}^{t}=v_{i}^{t-1}+\left(x_{i}^{t}-x_{*}\right)f,\\ x_{i}^{t}=x_{i}^{t-1}+v_{i}^{t}, \end{array}$$

where **x**_∗_ is the current global best location (solution) which is located after comparing all the solutions among all the *n* bats. In our experiments, we make *f* = 0.5. Through a series of simulation experiments on path planning for UCAV in Section 5.2, it was found that setting the parameter of pulse rate *r* to 0.6 and the loudness *A* to 0.95 produced the best results.

The second modification is to add mutation operator in an attempt to increase diversity of the population to improve the search efficiency and speed up the convergence to optima. For the local search part, once a solution is selected among the current best solutions, a new solution for each bat is generated locally using random walk by (8) when *ξ* is larger than pulse rate *r*, that is, *ξ* > *r*, where *ξ* ∈ [0, 1] is a random real number drawn from a uniform distribution; while when *ξ* ≤ *r*, we use mutation operator in DE updating the new solution to increase diversity of the population to improve the search efficiency by

(11) $$\begin{array}{c} x_{\text{new}}=x_{r_{1}}^{t}+F\left(x_{r_{2}}^{t}-x_{r_{3}}^{t}\right), \end{array}$$

where *F* is the mutation weighting factor, while *r*_1_, *r*_2_, and *r*_3_ are uniformly distributed random integer numbers between 1 and *NP*. Through testing on path planning for UCAV in Section 5.2, it was found that setting the parameter of mutation weighting factor *F* to 0.5 in (11) and scaling factor *ε* to 0.1 in (4) produced the best results.

Based on above-mentioned analyses, the mainframe of the bat algorithm with mutation (BAM) can be described as shown in Algorithm 3.

### 4.2. Algorithm BAM for UCAV Path Planning

BAM can adapt to the needs of UCAV path planning, while optimization algorithms can improve the BA fast search capabilities and increase the search to the global possible optimum solution. Fitness for bat *i* at position *x*_*i*_ is represented by the objective function shown as (4) in UCAV path planning model, the smaller the threat value, the lower the fitness for bat *i* at position *x*_*i*_.

Based on the above analysis, the pseudo code of improved BA-BAM for UCAV path planning is described as shown in Algorithm 4.

## 5. Simulation Experiments

In this section, we look at the performance of BAM as compared with other population-based optimization methods, such as ACO, BBO, DE, ES, GA, PBIL, PSO, and SGA. Firstly, we compare performances between BAM and other population-based optimization methods on the different parameters the maximum generation *Maxgen* and the dimension of converted optimization function *D*, and then we compare performances between BAM and BA on the different parameters loudness *A*, pulse rate *r*, weighting factor *F*, and scaling factor *ε* (where *F* and *ε* only for BAM).

To allow a fair comparison of running times, all the experiments were performed on a PC with an AMD Athlon(tm) 64 X2 Dual Core Processor 4200+ running at 2.20 GHz, 1024 MB of RAM, and a hard drive of 160 GB. Our implementation was compiled using MATLAB R2011b (7.13) running under Windows XP SP3. No commercial BBO tools or other population-based optimization tools were used in the following experiments.

### 5.1. General Performance of BAM

In this subsection, firstly we will present the supposed problem we use to test the performance of BAM. We use the parameters of battle field environments described as [1]. Supposed that there exists the following map information, UCAV flight from start point (10, 10) to end point (55, 100). In the flight course, there exist five threat areas. Their coordinates and corresponding threat radii are shown as in Table 1. Also, we set balanced coefficient between safety performance and fuel performance *k* = 0.5.

In order to explore the benefits of BAM, in this subsection we compared its performance on UCAV path planning problem with BA and eight other population-based optimization methods, which are ACO, BBO, DE, ES, GA, PBIL, PSO, and SGA. ACO (ant colony optimization) [20] is a swarm intelligence algorithm for solving computational problems which is based on the pheromone deposition of ants. Biogeography-based optimization (BBO) [21–23] is a new evolutionary algorithm (EA) developed for global optimization which is a generalization of biogeography to EA. DE (differential evolution) [14] is a simple but excellent optimization method that uses the difference between two solutions to probabilistically adapt a third solution. An ES (evolutionary strategy) [24] is an algorithm that generally distributes equal importance to mutation and recombination, and that allows two or more parents to reproduce an offspring. A GA (genetic algorithm) [25] is a search heuristic that mimics the process of natural evolution. PBIL (probability-based incremental learning) [26] is a type of genetic algorithm where the genotype of an entire population (probability vector) is evolved rather than individual members. PSO (particle swarm optimization) [18, 27] is also a swarm intelligence algorithm which is based on the swarm behavior of fish, and bird schooling in nature. A stud genetic algorithm (SGA) [28] is a GA that uses the best individual at each generation for crossover.

Except an ad hoc explain, in the following experiments, we use the same MATLAB code and parameters settings for other population-based optimization methods in [21, 29]. 

To compare the different effects among the parameters *Maxgen* and *D*, we ran 100 Monte Carlo simulations of each algorithm on the above UCAV path planning problem to get representative performances. For simplicity, we subtract 50 from the actual value; that is, if a value is 0.4419 in the following table, then its corresponding value 50.4419 is its true value. We must point out that we mark the best value with italic and bold font for each algorithm in Tables 2–5. 

#### 5.1.1. Effect of Maximum Generation: *Maxgen*

The choice of the best maximum generation of metaheuristic algorithm is always critical for specific problems. Increasing the maximum generation will increase the possibility of reaching optimal solution, promoting the exploitation of the search space. Moreover, the probability to find the correct search direction increases considerably. The influence of maximum generation is investigated in this sub-subsection. For all the population-based optimization methods, all the parameter settings are the same as above mentioned, only except for maximum generation *Maxgen* = 50, *Maxgen* = 100, *Maxgen* = 150, *Maxgen* = 200, and *Maxgen* = 250. The results are recorded in Tables 2, 3, 4, and 5 after 100 Monte Carlo runs.  Table 2 shows the best minima found by each algorithm over 100 Monte Carlo runs.  Table 3 shows the worst minima found by each algorithm over 100 Monte Carlo runs.  Table 4 shows the average minima found by each algorithm, averaged over 100 Monte Carlo runs.  Table 5 shows the average CPU time consumed by each algorithm, averaged over 100 Monte Carlo runs. In other words, Tables 2, 3, and 4 show the best, worst, and average performance of each algorithm, respectively, while Table 5 shows the average CPU time consumed by each algorithm.

From Table 2, we see that BAM performed the best on all the groups, while DE performed the second best on the 5 groups especially when *Maxgen* = 150, 200, and 250.  Table 3 shows that PBIL was the worst at finding objective function minima on all the five groups when multiple runs are made, while the BAM was the best on all the groups in the worst values.  Table 4 shows that BAM was the most effective at finding objective function minima when multiple runs are made, while DE and SGA performed the second best on the 5 groups, and GA and SGA similarly performed the third best on the 5 groups.  Table 5 shows that PBIL was the most effective at finding objective function minima when multiple runs are made, performing the best on all the 5 groups. By carefully looking at the results in Tables 2, 3, and 4, we can recognize that the values for each algorithm are obviously decreasing with the increasing *Maxgen*, while the performance of BAM increases little with the *Maxgen* increasing from 200 to 250, so we set *Maxgen* = 200 in other experiments. In sum, from Tables 2, 3, 4, and 5 we can draw the conclusion that the more the generations are, the smaller the objective function value we can reach, while the CPU time consumes more. Moreover, BAM performs better than other population-based optimization methods for the UCAV path planning problem with different maximum generation.

#### 5.1.2. Effect of Dimensionality: *D*

In order to investigate the influence of the dimension on the performance of BAM, we carry out a scalability study comparing with other population-based optimization methods for the UCAV path planning problem with the dimensionality *D* = 5, *D* = 10, *D* = 15, *D* = 20, *D* = 25, *D* = 30, *D* = 35, and *D* = 40. The results are recorded in Tables 6, 7, 8, and 9 after 100 Monte Carlo runs.  Table 6 shows the best minima found by each algorithm over 100 Monte Carlo runs.  Table 7 shows the worst minima found by each algorithm over 100 Monte Carlo runs.  Table 8 shows the average minima found by each algorithm, averaged over 100 Monte Carlo runs.  Table 9 shows the average CPU time consumed by each algorithm, averaged over 100 Monte Carlo runs. In other words, Tables 6, 7, and 8 show the best, worst, and average performance of each algorithm, respectively, while Table 9 shows the average CPU time consumed by each algorithm.

From Table 6, we see that DE performed the best when *D* = 10, while BAM performed the best on the other groups when multiple runs are made.  Table 7 shows that BA and ES were the worst when *D* = 5 and *D* = 10, respectively, and PBIL was the worst at finding objective function minima on all the other groups when multiple runs are made, while the DE, SGA, and GA were the best when *D* = 5, 10, and 15, respectively, and BAM was the best on the other groups in the worst values.  Table 8 shows that DE and SGA were the most effective when *D* = 5 and 10, respectively, and BAM was the best on the other groups at finding objective function minima when multiple runs are made.  Table 9 shows that PBIL was the most effective at finding objective function minima on all the groups. So, from the experimental results of this sub-subsection, we can conclude that the mutation operation between bats with a probability 1 − *r* during the process of generating new solutions has the ability to accelerate BA in general; especially the improvements are more significant at higher dimensionality. With the higher dimension, we are not always getting the better results with consuming more time; furthermore, the result is good enough when *D* = 20. In sum, in other experiments we should make *D* = 20 under the comprehensive consideration. 

### 5.2. Influence of Control Parameter

In [15], Yang concluded that if we adjust the parameters properly so that BA can outperform GA, HS (harmony search), and PSO. The choice of the control parameters is of vital importance for different problems. To compare the different effects among the parameters *A*, *r*, *F*, and *ε* (*F* and *ε* only for BAM), we ran 100 Monte Carlo simulations of BA and BAM algorithm on the above problem to get representative performances. 

#### 5.2.1. Loudness: *A*

To investigate the influence of the loudness on the performance of BAM, we carry out this experiment comparing BA for the UCAV path planning problem with the loudness *A* = 0, 0.1, 0.2, …, 0.9, 1.0 and fixed pulse rate *r* = 0.6. All other parameter settings are kept unchanged. The results are recorded in Tables 10, 11, 12, and 13 after 100 Monte Carlo runs.  Table 10 shows the best minima found by BA and BAM algorithms over 100 Monte Carlo runs.  Table 11 shows the worst minima found by BA and BAM algorithms over 100 Monte Carlo runs.  Table 12 shows the average minima found by BA and BAM algorithms averaged over 100 Monte Carlo runs.  Table 13 shows the average CPU time consumed by BA and BAM algorithms, averaged over 100 Monte Carlo runs. In other words, Tables 10, 11, and 12 show the best, worst, and average performance of BA and BAM algorithm, respectively, while Table 13 shows the average CPU time consumed by BA and BAM algorithms. 

From Table 10, we obviously see that BAM performed better (on average) than BA on all the groups, and BA and BAM reach the worst values 9.6473 and 11.9280 when *A* = 0, respectively, while BA and BAM reach the best values 4.0888 and 0.7774 when *A* = 1.0, respectively, among the optima when multiple runs are made.  Table 11 shows evidently that BAM performed better (on average) than BA on all the groups (except *A* = 0), and BA as well as BAM reach the worst values 39.0584 and 38.6449 when *A* = 0.1 and *A* = 0, respectively, while BA and BAM reach the best values 24.4673 and 8.8555 when *A* = 0.1 and *A* = 1.0, respectively, among the worst values when multiple runs are made.  Table 12 shows that BAM performed better (on average) than BA on all the groups, and BA and BAM reach the worst values 20.3072 and 20.2230 when *A* = 0, respectively, while BA and BAM reach the best values 11.1174 and 2.7086 when *A* = 1.0, respectively, among the mean values when multiple runs are made.  Table 13 shows that BA was more effective at finding objective function minima when multiple runs are made, performing the best on all the groups. By carefully looking at the results in Tables 10, 11, and 12, we can recognize that the threat value for BA and BAM is decreasing with the increasing *A*, and BA and BAM reach optima/minimum when *A* is equal or very close to 1.0, while BA and BAM reach maximum when *A* is equal or very close to 0. So, we set *A* = 0.95 which is very close to 1.0 in other experiments. In sum, from Tables 10, 11, 12, and 13, we can conclude that the mutation operation between bats during the process of the new solutions updating has the ability to accelerate BA in general.

#### 5.2.2. Pulse Rate: *r*

To investigate the influence of the pulse rate on the performance of BAM, we carry out this experiment comparing with BA for the UCAV path planning problem with the pulse rate *r* = 0, 0.1, 0.2, …, 0.9, 1.0 and fixed loudness *A* = 0.95. All other parameter settings are kept unchanged. The results are recorded in Tables 14, 15, 16, and 17 after 100 Monte Carlo runs.  Table 14 shows the best minima found by BA and BAM algorithms over 100 Monte Carlo runs.  Table 15 shows the worst minima found by BA and BAM algorithms over 100 Monte Carlo runs.  Table 16 shows the average minima found by BA and BAM algorithms, averaged over 100 Monte Carlo runs.  Table 17 shows the average CPU time consumed by BA and BAM algorithm, averaged over 100 Monte Carlo runs. In other words, Tables 14, 15, and 16 shows the best, worst, and average performance of BA and BAM algorithm respectively, while Table 17 shows the average CPU time consumed by BA and BAM algorithms. 

From Table 14, we obviously see that BAM performed better (on average) than BA on all the groups, and BA and BAM reach the worst values 5.1353 and 0.8536 when *r* = 1.0, respectively, while BA and BAM reach the best values 1.3626 and 0.4591 when *r* = 0.1 and *r* = 0.2, respectively, among the optima when multiple runs are made.  Table 15 shows evidently that BAM performed better (on average) than BA on all the groups, and BA and BAM reach the worst value 30.9979 and 12.3230 when *r* = 1.0 and *r* = 0.1, respectively, while BA and BAM reach the best values 17.8310 and 6.4524 when *r* = 0.2 and *r* = 0.7, respectively, among the worst values when multiple runs are made.  Table 16 shows that BAM performed better (on average) than BA on all the groups, and BA and BAM reach the worst mean values 11.1155 and 2.9290 when *r* = 1.0, respectively, while BA and BAM reach the best mean values 6.8803 and 0.7729 when *r* = 0.6, respectively, among the mean values when multiple runs are made.  Table 13 shows that BA was more effective at finding objective function minima when multiple runs are made, performing the best on all the groups. By carefully looking at the results in Tables 14, 15, and 16, we can recognize that the threat value for BA and BAM varies little with the increasing *A*, and BA and BAM reach mean optima/minima when *r* is equal or very close to 0.6, while BA and BAM reach maximum when *r* is equal or very close to 1.0. So, we set *r* = 0.6 in other experiments. In sum, from Tables 14, 15, 16, and 17 we can conclude that the mutation operation between bats during the process of generating new solutions has the ability to accelerate BA in general.

#### 5.2.3. Weighting Factor: *F*

For the sake of investigating the influence of the weighting factor *F* on the performance of BAM, we carry out this experiment for the UCAV path planning problem with the weighting factor *F* = 0, 0.1, 0.2, …, 1.5 and fixed scaling factor *ε* = 0.1. All other parameter settings are kept unchanged. The results are recorded in Table 18 after 100 Monte Carlo runs. Columns 1, 2, and 3 in Table 18 show the best, worst, and average performances of BAM algorithm, respectively, while Column 4 in Table 18 shows the average CPU time consumed by BAM algorithm.

From Table 18, we can recognize that the threat values for BAM varies little with the increasing *F*, and BAM reaches optimum/minimum on *F* = 0.5. So, we set *F* = 0.5 in other experiments. From Table 18 we can draw the conclusion that BAM is insensitive to the weighting factor *F*, so we do not have to fine-tune the parameter *F* to get the best performance for different problems.

#### 5.2.4. Scaling Factor: ***ε***

For the sake of investigating the influence of the scaling factor *ε* on the performance of BAM, we carry out this experiment for the UCAV path planning problem with the factor scaling factor *ε* = 0, 0.1, 0.2, …, 1.0 and fixed weighting factor *F* = 0.5. All other parameter settings are kept unchanged. The results are recorded in Table 19 after 100 Monte Carlo runs. Columns 1, 2, and 3 in Table 19 shows the best, worst, and average performances of BAM algorithms respectively, while Column 4 in Table 19 shows the average CPU time consumed by BAM algorithm.

From Table 19, we can recognize that the values for BAM vary little with the increasing *ε*, and BAM reaches optimum/minimum and the worst/maximum on *ε* = 0.1 and *ε* = 0, respectively. So, we set *ε* = 0.1 in other experiments. From Table 19 we can draw the conclusion that BAM is insensitive to the scaling factor *ε*, so we do not have to fine-tune the parameter *ε* to get the best performance for different problems.

The simulation experiment performed in Sections 5.1 and 5.2 shows that the algorithm BAM we proposed performed the best but worst effectively when solving the UCAV path planning problem. From deep investigation, we can see that BAM cam reach minima when maximum generation *Maxgen* = 50 and population size Popsize = 30, while other population-based optimization methods cannot achieve satisfactory result under this condition; that is, BAM needs fewer maximum generation, less population size, and less time than other population-based optimization methods when arriving to the same performance. In sum, the simulation implemented in Section 6 shows that the algorithm BAM we proposed performed the best and most absolutely effectively, and it can solve the UCAV path planning problem perfectly. Furthermore, comparing to other population-based optimization methods, the algorithm BAM is insensitive to the parameter loudness*A*, pulse rate *r*, weighting factor *F*, and scaling factor *ε*, so we do not have to fine-tune the parameters *A*, *r*, *F*, and *ε* to get the best performance for different problems.

### 5.3. Discussions

The BA algorithm is a simple, fast, and robust global optimization algorithm developed by X. S. Yang in 2010. However, it may lack the diversity of population between bats. Therefore, in this work, we add mutation operation between bats to the BA during the process of new solutions updating. And then, the BAM algorithm is proposed to solve the UCAV path planning. From the experimental results we can sum up the following:

- Our proposed BAM approach is effective and efficient. It can solve the UCAV path planning problem effectively.
- The overall performance of BAM is superior to or highly competitive with BA and other compared state-of-the-art population-based optimization methods.
- BAM and other population-based optimization methods were compared for different maximum generations and the dimension. Under majority conditions, BAM is significantly substantial better than other population-based optimization methods.
- BAM and BA were compared for different loudness *A* and pulse rate *r*, weighting factor *F*, and scaling factor *ε*. Under almost all the conditions, BAM is far better than BA.
- The algorithm BAM is insensitive to the parameter loudness *A* and discovery rate *r*, weighting factor *F*, and scaling factor *ε*, so we do not have to fine-tune the parameters *A*, *r*, *F*, and *ε* to get the best performance for different problems.

## 6. Conclusion and Future Work

This paper presented a bat algorithm with mutation for UCAV path planning in complicated combat field environments. A novel type of BA model has been described for single UCAV path planning, and a modification is applied to mutate between bats during the process of the new generation generating. Then, the UCAV can find the safe path by connecting the chosen nodes while avoiding the threat areas and costing minimum fuel. This new approach can accelerate the global convergence speed while maintaining the strong robustness of the basic BA. The detailed implementation procedure for this improved metaheuristic approach is also described. Compared with other population-based optimization methods, the simulation experiments show that this improved method is a feasible and effective way in UCAV path planning. It is also flexible, in complicated dynamic battle field environments and pop-up threats are easily incorporated. 

In the algorithm of UCAV path planning, there are many issues worthy of further study, and efficient route planning method should be developed depending on the analysis of specific combat field environments. Currently, the hot issue contains self-adaptive route planning for a single UCAV and collaborative route planning for a fleet of UCAVs. As the important ways of improving aircraft survivability, adaptive route planning should analyze real-time data under the uncertain and dynamic threat condition; even it can re-modify preplanned flight path to improve the success rate of completing mission. The difficulty of the collaborative route planning for a fleet of UCAVs exists in coordination between the various UCAVs, including the fleet formation, target distribution, arrival time constraint, and avoidance conflict, each of which is a complicated question worthy of further study. Our future work will focus on the two hot issues and develop new methods to solve problem in UCAV path planning and replanning.

## Figures and Tables

**Algorithm 1:**
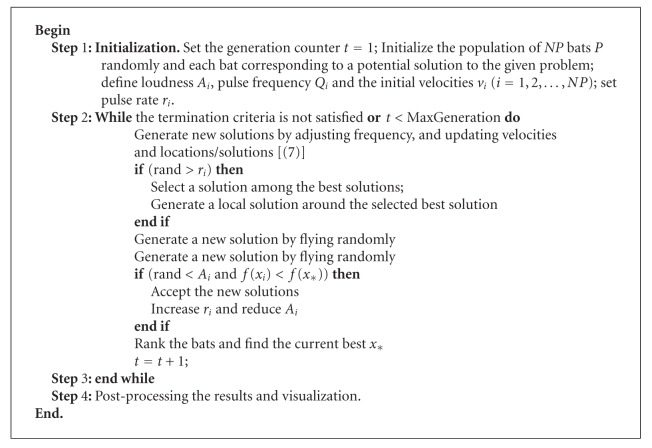
Bat Algorithm.

**Algorithm 2:**
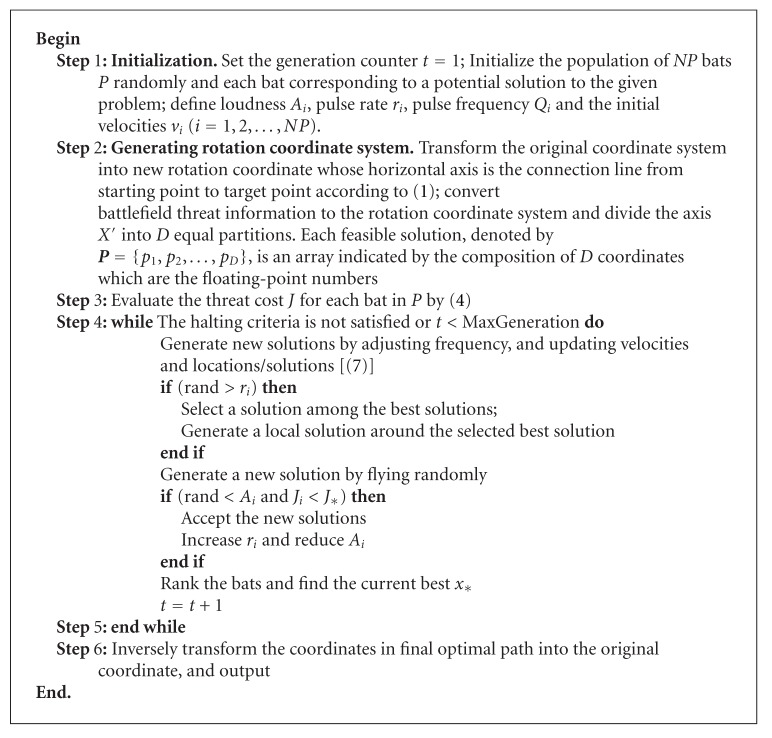
Algorithm of BA for UCAV path planning.

**Algorithm 3:**
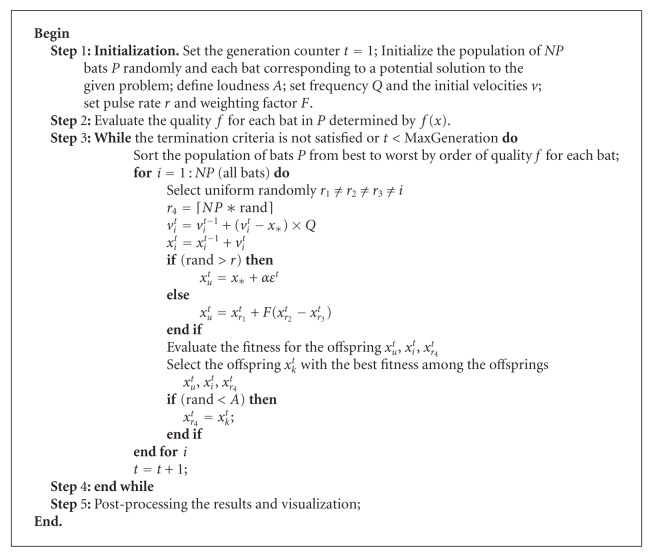
Bat algorithm with mutation.

**Algorithm 4:**
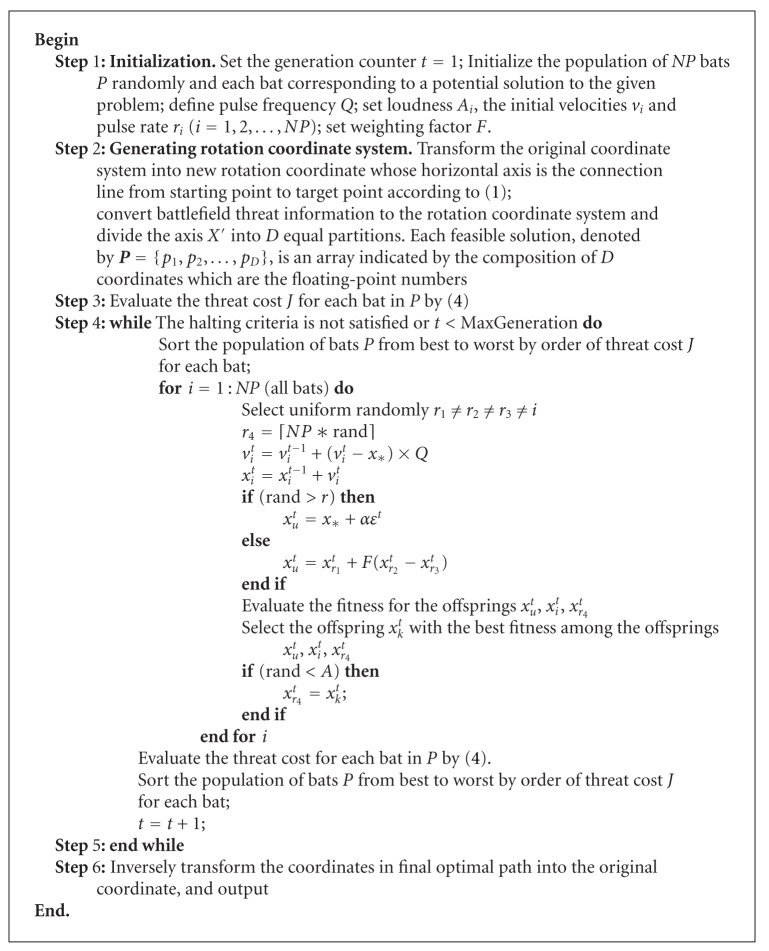
Algorithm of BAM for UCAV path planning.

**Table 1:** Information about known threats.

No.	Location (km)	Threat radius (km)	Threat grade
1	(45,50)	10	2
2	(12,40)	10	10
3	(32,68)	8	1
4	(36,26)	12	2
5	(55,80)	9	3

**Table 2:** Best normalized optimization results on UCAV path planning problem on different *Maxgen*. The numbers shown are the best results found after 100 Monte Carlo simulations of each algorithm.

Parameter			Algorithm									
Popsize	*Maxgen *	*D *	ACO	BA	BAM	BBO	DE	ES	GA	PBIL	PSO	SGA
30	**50**	20	10.7202	4.0662	**0.6208**	7.0272	2.4179	9.6276	1.2604	100.0527	2.7827	1.7370
30	**100**	20	10.8912	4.7582	**0.4900**	4.7484	0.8503	10.6318	1.5073	98.3640	2.3469	1.3218
30	**150**	20	9.9096	4.1112	**0.4724**	4.2311	0.5319	11.1469	1.0991	71.2093	2.3738	1.1559
30	**200**	20	12.3080	3.1463	**0.4590**	2.7765	0.5047	11.2403	1.0792	72.8244	3.4276	0.7595
30	**250**	20	7.1358	4.4072	**0.4636**	2.6109	0.4792	12.3745	1.0640	74.9071	2.5221	1.0166

**Table 3:** Worst normalized optimization results on UCAV path planning problem on different *Maxgen*. The numbers shown are the worst results found after 100 Monte Carlo simulations of each algorithm.

Parameter			Algorithm									
Popsize	*Maxgen *	*D*	ACO	BA	BAM	BBO	DE	ES	GA	PBIL	PSO	SGA
30	**50**	20	18.7099	39.0832	**11.7494**	30.2785	25.3999	41.9676	10.2501	**464.2014**	28.6115	13.0102
30	**100**	20	18.4316	29.9962	**9.7666**	32.1868	18.6288	38.5875	8.2047	**339.5171**	25.7065	11.0529
30	**150**	20	17.4223	31.1293	**7.6952**	29.5695	13.8150	46.0828	10.5257	**457.5577**	29.6341	13.3517
30	**200**	20	17.2147	24.9732	**6.7334**	41.5292	10.4226	31.3944	6.7466	**308.6347**	33.0709	7.5385
30	**250**	20	16.9896	24.7175	**3.3564**	19.5894	8.9560	34.8908	8.9162	**201.3705**	27.3858	13.5830

**Table 4:** Mean normalized optimization results on UCAV path planning problem on different *Maxgen*. The numbers shown are the minimum objective function values found by each algorithm, averaged over 100 Monte Carlo simulations.

Parameter			Algorithm									
Popsize	*Maxgen *	D	ACO	BA	BAM	BBO	DE	ES	GA	PBIL	PSO	SGA
30	**50**	20	16.3819	16.6782	**1.4842**	14.1072	12.3797	20.5653	4.2541	219.9368	10.0760	4.5491
30	**100**	20	16.2884	14.9048	**0.9337**	13.5705	6.0887	20.6706	3.5523	166.4567	9.1725	3.4353
30	**150**	20	16.1408	14.4874	**0.9123**	11.7978	3.7267	20.1996	3.4269	142.1862	9.5459	3.1636
30	**200**	20	16.3976	12.4323	**0.8000**	11.8224	2.6358	20.8610	3.0080	131.1306	8.9917	2.6434
30	**250**	20	16.1958	11.4213	**0.7422**	10.0553	1.9715	20.7600	2.9160	119.6745	7.8005	3.1409

**Table 5:** Average CPU time on UCAV path planning problem on different *Maxgen*. The numbers shown are the minimum average CPU time (sec) consumed by each algorithm.

Parameter			Algorithm									
Popsize	*Maxgen *	D	ACO	BA	BAM	BBO	DE	ES	GA	PBIL	PSO	SGA
30	**50**	20	1.1477	1.2389	2.5415	0.7540	1.0830	1.1045	1.0068	**0.5610**	0.9389	0.9733
30	**100**	20	2.2752	2.5180	5.0720	1.5041	2.1782	2.2028	1.9875	**1.0793**	1.8632	1.9253
30	**150**	20	3.4043	3.7411	7.3826	2.2564	3.2397	3.2778	2.9604	**1.5839**	2.7619	2.8612
30	**200**	20	4.5337	4.9930	9.7353	3.0201	4.3053	4.3581	3.9755	**2.0459**	3.6100	3.7132
30	**250**	20	5.6563	6.1668	12.2422	3.6952	5.4137	5.3999	4.9278	**2.6083**	4.6532	4.7580

**Table 6:** Best normalized optimization results on UCAV path planning problem on different D. The numbers shown are the best results found after 100 Monte Carlo simulations of each algorithm.

Parameter			Algorithm									
Popsize	*Maxgen *	D	ACO	BA	BAM	BBO	DE	ES	GA	PBIL	PSO	SGA
30	200	**5**	10.1164	10.6909	**4.3575**	10.2341	4.3568	12.3746	5.2471	8.5576	5.6082	9.9596
30	200	**10**	7.4746	2.3600	1.3953	2.6157	**1.3952**	8.0656	1.5716	25.6821	2.1101	1.5498
30	200	**15**	9.8297	3.0757	**0.6094**	2.0896	0.6204	7.7408	0.8299	61.4656	3.2257	0.9700
30	200	**20**	10.0836	2.3950	**0.4679**	2.7765	0.4913	9.6276	0.8600	72.2897	2.3738	0.8426
30	200	**25**	11.5490	5.0173	**0.4484**	4.8474	0.6265	12.3169	1.5243	113.7537	2.3740	1.3743
30	200	**30**	13.8615	7.2470	**0.4671**	10.9403	1.1301	18.0090	1.7026	152.0173	3.6751	1.5147
30	200	**35**	16.9476	7.4484	**0.4795**	10.4147	1.2849	16.8613	2.1602	254.0060	5.4765	1.5319
30	200	**40**	17.6142	8.6500	**0.6028**	14.4997	3.9617	19.8244	2.4178	315.4459	5.5384	1.9406

**Table 7:** Worst normalized optimization results on UCAV path planning problem on different D. The numbers shown are the worst results found after 100 Monte Carlo simulations of each algorithm.

Parameter			Algorithm									
Popsize	*Maxgen *	D	ACO	BA	BAM	BBO	DE	ES	GA	PBIL	PSO	SGA
30	200	**5**	12.6928	**295.2557**	10.2403	119.9434	**9.7959**	62.1765	20.1888	22.9251	13.3267	22.6326
30	200	**10**	18.2565	58.7386	10.7242	42.1924	12.4821	**74.6665**	6.3799	64.0778	23.2604	**5.7899**
30	200	**15**	10.9917	35.7454	10.1928	34.8307	12.5250	50.3214	**8.1499**	**119.2700**	28.0228	9.9385
30	200	**20**	17.0266	33.7068	**3.7420**	32.1908	18.8897	38.7234	9.4820	**254.0913**	34.7133	11.6024
30	200	**25**	12.2373	24.9265	**3.5192**	30.9943	17.1415	33.4598	12.7971	**593.9572**	31.6741	16.0736
30	200	**30**	14.4647	30.0844	**10.2851**	61.7204	29.6529	37.4566	22.1291	**2011**	35.6656	14.0512
30	200	**35**	18.7271	32.7374	**8.8193**	37.9424	39.4435	46.6475	24.4790	**8424.28**	38.0578	15.6693
30	200	**40**	27.0641	33.2634	**8.4273**	49.5461	45.4130	44.3624	19.2098	**8856.06**	35.5090	22.5022

**Table 8:** Mean normalized optimization results on UCAV path planning problem on different D. The numbers shown are the minimum objective function values found by each algorithm, averaged over 100 Monte Carlo simulations.

Parameter			Algorithm									
Popsize	*Maxgen *	D	ACO	BA	BAM	BBO	DE	ES	GA	PBIL	PSO	SGA
30	200	**5**	11.4856	56.4830	9.0542	23.4238	**8.0557**	31.8202	10.5709	15.5053	10.0765	10.8836
30	200	**10**	12.5333	19.4251	2.7075	8.7776	3.1206	27.2252	2.3722	51.2935	7.2212	**2.2813**
30	200	**15**	10.2484	13.6018	**1.2318**	8.9120	2.3737	22.0792	2.1136	78.4948	7.7362	1.8973
30	200	**20**	16.3303	13.6305	**0.7609**	12.2883	3.0044	20.4717	2.9612	127.5765	9.9091	2.8621
30	200	**25**	11.5842	14.9017	**0.7093**	15.3698	4.6029	22.7244	3.7244	214.0821	10.3315	3.7238
30	200	**30**	13.9422	16.6162	**1.1067**	18.6997	11.4103	25.4016	5.3097	335.0904	12.7964	4.3798
30	200	**35**	18.3452	17.7033	**1.4617**	20.7753	19.1074	27.2172	6.0765	661.1281	13.8799	5.4943
30	200	**40**	24.7642	19.9737	**1.8769**	25.9148	28.7062	30.0177	7.6989	1174.90	15.1555	7.4237

**Table 9:** Average CPU time on UCAV path planning problem on different D. The numbers shown are the minimum average CPU time (sec) consumed by each algorithm.

Parameter			Algorithm									
Popsize	*Maxgen *	D	ACO	BA	BAM	BBO	DE	ES	GA	PBIL	PSO	SGA
30	200	**5**	1.93	1.87	3.58	1.26	2.03	2.13	2.21	**1.19**	2.27	2.12
30	200	**10**	2.86	3.08	5.28	1.86	2.64	2.88	2.83	**1.46**	2.79	2.81
30	200	**15**	3.61	3.92	7.61	2.31	3.47	3.60	3.43	**1.76**	3.31	3.34
30	200	**20**	4.50	4.95	9.83	3.02	4.24	4.33	3.93	**2.10**	3.73	3.84
30	200	**25**	5.57	5.83	12.06	3.37	5.00	4.96	4.43	**2.43**	4.22	4.39
30	200	**30**	6.44	7.30	14.40	3.86	5.58	5.84	4.91	**2.75**	4.70	4.90
30	200	**35**	7.30	8.39	16.84	4.46	6.23	6.63	5.65	**3.14**	5.16	5.39
30	200	**40**	8.34	9.60	19.34	4.97	6.71	7.34	6.06	**3.38**	5.68	5.96

**Table 10:** Best normalized optimization results on UCAV path planning problem on different A. The numbers shown are the best results found after 100 Monte Carlo simulations of BA and BAM algorithms.

Parameter		Algorithm	
A	*r *	BA	BAM
**0**	0.6	**9.6473**	**11.9280**
**0.1**	0.6	6.6352	2.3678
**0.2**	0.6	6.9462	1.9257
**0.3**	0.6	5.6720	1.2626
**0.4**	0.6	8.5845	1.0167
**0.5**	0.6	4.3351	1.1764
**0.6**	0.6	5.8639	0.9442
**0.7**	0.6	5.5337	0.9305
**0.8**	0.6	5.2613	0.9942
**0.9**	0.6	5.3534	1.0012
**1.0**	0.6	**4.0888**	**0.7774**

**Table 11:** Worst normalized optimization results on UCAV path planning problem on different A. The numbers shown are the worst results found after 100 Monte Carlo simulations of BA and BAM algorithms.

Parameter		Algorithm	
A	*r *	BA	BAM
**0**	0.6	33.4435	**38.6449**
**0.1**	0.6	**39.0584**	20.9711
**0.2**	0.6	38.7234	14.8265
**0.3**	0.6	29.4638	12.6085
**0.4**	0.6	36.3300	12.8931
**0.5**	0.6	26.0305	8.9874
**0.6**	0.6	28.7598	13.6922
**0.7**	0.6	26.2501	11.0757
**0.8**	0.6	**24.4673**	12.6789
**0.9**	0.6	29.4221	9.1986
**1.0**	0.6	25.7065	**8.8555**

**Table 12:** Mean normalized optimization results on UCAV path planning problem on different A. The numbers shown are the minimum objective function values found by BA and BAM algorithms, averaged over 100 Monte Carlo simulations.

Parameter		Algorithm	
A	*r *	BA	BAM
**0**	0.6	**20.3072**	**20.2230**
**0.1**	0.6	18.3087	9.5999
**0.2**	0.6	16.7149	5.8455
**0.3**	0.6	14.5081	4.5573
**0.4**	0.6	15.2639	4.1528
**0.5**	0.6	13.3459	3.5631
**0.6**	0.6	12.6488	3.5585
**0.7**	0.6	12.6416	3.2400
**0.8**	0.6	11.9422	3.2585
**0.9**	0.6	12.5489	2.7930
**1.0**	0.6	**11.1174**	**2.7086**

**Table 13:** Average CPU time on UCAV path planning problem on different A. The numbers shown are the minimum average CPU time (Sec) consumed by BA and BAM algorithms.

Parameter		Algorithm	
A	*r *	BA	BAM
**0**	0.6	**4.81**	**9.77**
**0.1**	0.6	4.85	**8.75**
**0.2**	0.6	4.82	8.87
**0.3**	0.6	4.85	9.01
**0.4**	0.6	**4.88**	9.18
**0.5**	0.6	**4.88**	9.37
**0.6**	0.6	4.84	9.19
**0.7**	0.6	4.81	9.39
**0.8**	0.6	4.85	9.43
**0.9**	0.6	4.86	9.52
**1.0**	0.6	**4.88**	9.47

**Table 14:** Best normalized optimization results on UCAV path planning problem on different r. The numbers shown are the best results found after 100 Monte Carlo simulations of BA and BAM algorithms.

Parameter		Algorithm	
A	*r *	BA	BAM
0.5	**0**	1.9003	0.4709
0.5	**0.1**	**1.3626**	0.4598
0.5	**0.2**	2.4637	**0.4591**
0.5	**0.3**	1.7444	0.4669
0.5	**0.4**	1.7388	0.4633
0.5	**0.5**	2.6682	0.4600
0.5	**0.6**	2.4835	0.4614
0.5	**0.7**	1.8795	0.4702
0.5	**0.8**	2.2624	0.4711
0.5	**0.9**	3.5876	0.5019
0.5	**1.0**	**5.1353**	**0.8536**

**Table 15:** Worst normalized optimization results on UCAV path planning problem on different r. The numbers shown are the worst results found after 100 Monte Carlo simulations of BA and BAM algorithms.

Parameter		Algorithm	
A	*r *	BA	BAM
0.95	**0**	29.4969	7.5532
0.95	**0.1**	25.5864	**12.3230**
0.95	**0.2**	**17.8310**	9.2830
0.95	**0.3**	30.4250	10.7164
0.95	**0.4**	21.2176	10.1811
0.95	**0.5**	20.8479	9.0701
0.95	**0.6**	17.8944	7.0315
0.95	**0.7**	28.7659	**6.4524**
0.95	**0.8**	28.7659	9.3845
0.95	**0.9**	21.9160	7.4771
0.95	**1.0**	**30.9979**	9.3678

**Table 16:** Mean normalized optimization results on UCAV path planning problem on different r. The numbers shown are the minimum objective function values found by BA and BAM algorithms, averaged over 100 Monte Carlo simulations.

Parameter		Algorithm	
A	*r *	BA	BAM
0.95	**0**	9.9001	0.8457
0.95	**0.1**	9.0247	1.0440
0.95	**0.2**	7.5435	1.0412
0.95	**0.3**	8.0903	1.0977
0.95	**0.4**	7.2892	0.8794
0.95	**0.5**	7.1919	0.9456
0.95	**0.6**	**6.8803**	**0.7729**
0.95	**0.7**	7.3384	0.8632
0.95	**0.8**	7.8609	0.8817
0.95	**0.9**	9.0251	1.1881
0.95	**1.0**	**11.1155**	**2.9290**

**Table 17:** Average CPU time on UCAV path planning problem on different r. The numbers shown are the minimum average CPU time (sec) consumed by BA and BAM algorithms.

Parameter		Algorithm	
*A *	r	BA	BAM
0.95	**0**	5.02	**9.90**
0.95	**0.1**	4.96	9.88
0.95	**0.2**	5.03	9.83
0.95	**0.3**	4.95	9.76
0.95	**0.4**	4.99	**9.90**
0.95	**0.5**	**5.08**	9.83
0.95	**0.6**	5.06	9.86
0.95	**0.7**	5.11	9.82
0.95	**0.8**	4.97	9.79
0.95	**0.9**	5.00	9.66
0.95	**1.0**	**4.87**	**9.43**

**Table 18:** Best normalized optimization results and average CPU time on UCAV path planning problem on different F. The numbers shown are the best results found after 100 Monte Carlo simulations of BAM algorithm.

Parameter		Algorithm			
F	*ε*	BAM			
		Best	Worst	Mean	CPU time (Sec)
**0**	0.1	0.7045	9.6041	**2.1675**	9.58
**0.1**	0.1	0.7806	11.3137	1.7250	9.69
**0.2**	0.1	**0.8138**	11.0360	1.8369	**9.75**
**0.3**	0.1	0.7421	9.1672	1.6696	9.72
**0.4**	0.1	0.6879	9.2307	1.6681	9.74
**0.5**	0.1	0.6843	9.1385	**1.5362**	9.78
**0.6**	0.1	0.6903	7.9582	1.9313	9.61
**0.7**	0.1	0.7193	7.8036	1.8229	9.65
**0.8**	0.1	0.6645	9.5210	2.0243	9.57
**0.9**	0.1	0.6964	9.3858	1.8923	9.58
**1.0**	0.1	0.7546	9.5856	1.8489	9.73
**1.1**	0.1	**0.6331**	**13.0251**	1.7814	9.72
**1.2**	0.1	0.6508	9.0815	1.6803	9.74
**1.3**	0.1	0.6694	9.9523	1.9246	**9.53**
**1.4**	0.1	0.6966	8.3557	1.6807	9.74
**1.5**	0.1	0.7112	**7.7598**	1.8652	9.62

**Table 19:** Best normalized optimization results and average CPU time on UCAV path planning problem on different ε. The numbers shown are the best results found after 100 Monte Carlo simulations of BAM algorithm.

Parameter		Algorithm			
*F *	*ε*	BAM			
		Best	Worst	Mean	CPU Time (sec)
0.5	**0**	**0.8817**	**12.3484**	**2.9293**	**9.08**
0.5	**0.1**	**0.4541**	**3.4890**	**0.7290**	**9.84**
1.0	**0.2**	0.4860	6.7479	0.9062	9.77
0.5	**0.3**	0.5158	7.8852	1.0775	9.76
0.5	**0.4**	0.5580	7.9747	1.0161	9.76
0.5	**0.5**	0.5570	8.6947	1.4135	9.67
0.5	**0.6**	0.6282	10.1393	1.3083	9.80
0.5	**0.7**	0.6363	10.8901	1.2981	9.82
0.5	**0.8**	0.6442	7.6385	1.5515	9.74
0.5	**0.9**	0.6779	9.9564	1.8094	9.68
0.5	**1.0**	0.7060	9.5250	1.9116	9.61
